# Sex differences in the association between blood pressure and atrial fibrillation: A case-control study

**DOI:** 10.3389/fcvm.2022.1061240

**Published:** 2022-12-08

**Authors:** Xiexiong Zhao, Qilun Feng, Abdul Wahid, Xiaoyan Wang, Juan Wen, Weihong Jiang, Xiaohong Tang

**Affiliations:** ^1^Department of Cardiology, The Third Xiangya Hospital, Central South University, Changsha, China; ^2^Department of Cardiology, The Affiliated Nanhua Hospital, University of South China, Hengyang, China

**Keywords:** hypertension, blood pressure, atrial fibrillation, sex differences, case control study

## Abstract

**Background:**

To examine the association of hypertension (HBP) and its control with atrial fibrillation (AF) and how patient sex affects this association.

**Materials and methods:**

A case control study of patients admitted to our hospital from 2015 to 2019 was conducted. Patients were divided into subgroups according to their blood pressure (BP) levels and control status, in which odd ratios (OR) by sex for AF was estimated using a logistic regression model and restrictive cubic splines before and after propensity score matching.

**Results:**

A total of 3,212 patients with AF and 8,307 without AF were investigated. Compared to patients with normal BP, patients with HBP had more AF [OR = 1.75 (1.52–2.02), OR = 2.66 (2.24–3.15), and OR = 4.30 (3.40–5.44) in patients with grade 1, 2, and 3 HBP, respectively]. In HBP patients with grade 3, the OR of AF was much higher in women than in men (OR = 7.15, 95% CI: 4.43–11.50 vs. OR = 2.48, 95% CI: 1.66–3.72). BP over 133.1/79.9 mmHg in men or 127.1/75.1 mmHg in women was positively associated with AF. In patients with HBP, uncontrolled BP was more associated with AF (OR = 3.00, 95% CI: 2.53–3.56), especially in women (OR = 3.09, 95% CI: 2.27–4.19). BP and prevalence of AF correlated with each other positively in patients admitted to a cardiology ward. Lowering BP to 145.1/85.8 mmHg in men or 140.5/82.5 mmHg in women led to less AF.

**Conclusion:**

There is more significant relationship between HBP and AF in female patients. A lower and individualized BP target may be formulated to prevent AF in women.

## Introduction

Arterial hypertension (HBP) and atrial fibrillation (AF) are closely related and often coexist clinically (1). About 60–80% of AF patients have HBP (2), and persistently elevated blood pressure (BP) is one of the important risk factors for AF (3–5). Conversely, patients with HBP are about several times more likely to develop AF than those with normal BP (6, 7). Studies have also shown that high normal BP increases the risk of AF in middle-aged individuals (6, 8).

However, it is uncertain above which BP level the risk of AF is significantly increased. Also, the extent to which BP control reduces AF in hypertensive patients is unknown, despite clear evidence that BP control reduces target organ damage and improves overall survival (9–11). Though some researches demonstrated an ideal BP in patients with AF (12), most current HBP guidelines do not specify BP-lowering goals for patients with AF (13–16). Considering that the target organ damage in HBP patients with AF is significantly more severe than that in patients with HBP alone (17), it is of great significance to establish BP control targets to reduce the risk of AF in HBP patients.

In many diseases, clinical manifestations vary by sex. Specifically, females have a lower overall rate of HBP than males (13), but post-menopausal females experience a faster rise in BP (18). On the other hand, AF is more common in males (19). However, few studies have explored sex differences in the relationship between BP and AF.

In this study, we aimed to examine in detail the association of BP and its control status with AF using a large case-control patient population. We also investigated sex differences in the relationship of BP and AF to provide more personalized suggestions for clinical treatment.

## Materials and methods

### Study population

**FIGURE 1:**
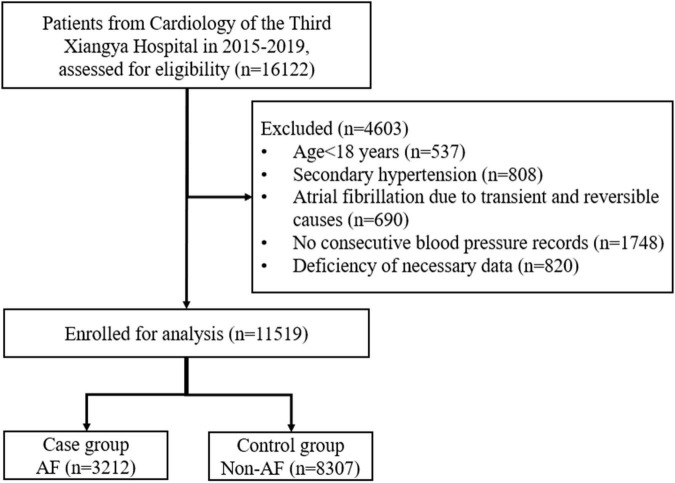
Flow chart of screening. AF, atrial fibrillation.

All patients admitted to the Cardiology Department of the Third Xiangya Hospital of Central South University during 2015–2019 were eligible. Among them, we included patients who were (a) ≥ 18 years of age and (b) had BP recorded at least daily for three consecutive days after admission. We excluded those diagnosed with (a) secondary HBP and (b) AF due to transient and reversible causes (e.g., infection, acute myocardial infarction, acute myocarditis, and hyperthyroidism). Those missing necessary data were also excluded. The included patients were assigned to case group or control group according to the presence or absence of AF. A flowchart for screening patients for analysis is shown in Figure 1. This article was organized in agreement with the STrengthening the Reporting of OBservational studies in Epidemiology (STROBE) statement (20).

### Data collection

We collected basic patient characteristics (i.e., unique identification number, sex, age, height, and weight), medical history (i.e., smoking, alcohol drinking, HBP, AF, diabetes, stroke, coronary artery disease, heart failure, valvular heart disease, cardiomyopathy, chronic obstructive pulmonary disease (COPD), and renal insufficiency), BP, and heart rate (HR). For patients with two or more admissions, only the data from the last admission were collected for analysis.

### Definition of parameters

#### Blood pressure and heart rate

Blood pressure (BP) and HR were measured by professional nurses or doctors using the Omron HBP-1300. The BP monitoring device was calibrated annually. The BP and HR records were obtained from the Hospital Information System (HIS), and the average of the BP and HR records for three consecutive days after admission was calculated as the final BP and HR for analysis.

#### Atrial fibrillation

A patient is considered to have a history of AF if he/she had a clear history of AF or if the electrocardiogram showed AF during hospitalization, but was excluded if the exclusive criteria were met.

#### Hypertension

A patient is considered to have a history of HBP if he/she had a clear history of HBP or if BP monitoring during hospitalization met the diagnostic criteria for HBP (including office BP or ambulatory BP).

#### Grade of blood pressure

Grade 1 is systolic and diastolic BP (SBP/DBP) ≥ 140/90 mmHg and < 160/100 mmHg; Grade 2 is SBP/DBP ≥ 160/100 mmHg and < 180/110 mmHg; and Grade 3 is SBP/DBP ≥ 180/110 mmHg.

#### Blood pressure uncontrolled

A patient with a history of HBP is considered BP controlled if BP was < 140/90 mmHg at the time of admission after lifestyle adjustment or medication; Otherwise, the patient is considered BP uncontrolled.

#### Valvular heart disease

Refers to moderate to severe mitral stenosis, mitral insufficiency, aortic stenosis, and aortic insufficiency.

#### Cardiomyopathy

Includes dilated cardiomyopathy, hypertrophic cardiomyopathy, restrictive cardiomyopathy, myocardial amyloidosis, and other confirmed cardiomyopathies.

#### Renal insufficiency

Estimated glomerular filtration rate (eGFR) < 60 ml/min/1.73 m^2^.

### Statistical analysis

We used the independent *t*-test to compare continuous variables and the Chi-square test (χ^2^) to compare categorical variables. Odd ratios (OR) between AF and Non-AF groups were analyzed using logistic regression and restricted cubic splines (RCS).

To address the confounding of patient demographics in this observational study, we estimated propensity scores for the likelihood of developing AF, and based on the estimated propensity scores, we matched patients with AF with those without AF using a 1:2 ratio without replacement (21). We then assessed the relationship between HBP and AF as well as sex differences in the relationship using data before and after propensity score matching (PSM).

For patients with or without AF, we used propensity score models adjusted for the following variables–sex, age, BMI, HR, smoking, alcohol drinking, diabetes, stroke, coronary artery disease, heart failure, valvular heart disease, cardiomyopathy, COPD, and renal insufficiency. PSM was implemented using a nearest-neighbor strategy. If the unspecified approach did not produce a satisfactory balance, the caliper for the matching was specified in the nearest-neighbor strategy. We then estimated comparative AF-vs.-Non AF ORs using conditional logistic regression and RCS.

Analyses were performed using RStudio version 2022.02.3 (with the packages MatchIt and rms). A *P*-value less than 0.05 was considered significant.

### Ethics statement

The Institutional Review Board of the third Xiangya Hospital approved the waiver of informed consent (No. I22317). All data were collected anonymously from the HIS and all authors ensure the confidentiality of patient data.

## Results

### Clinical characteristics

Of the 16,122 hospitalized patients, 11,519 were included in the study for further analysis after excluding those who met the exclusion criteria. Among these included patients, 3,212 were assigned to the case group (with AF) and 8,307 to the control group (without AF) (Figure 1).

Compared to controls without AF, the AF case group had significantly higher proportions of the following patient variables: female (47.8 vs. 45.4%, *P* = 0.025), HBP (75.8 vs. 61.4%, *P* < 0.001), smoking (27.9 vs. 25.3%, *P* = 0.006), diabetes (21.0 vs. 17.6%, *P* < 0.001), stroke (16.3 vs. 11.0%, *P* < 0.001), coronary artery disease (29.5 vs. 24.4%, *P* < 0.001), heart failure (27.8 vs. 21.3%, *P* < 0.001), valvular heart disease (10.0 vs. 6.7%, *P* < 0.001), cardiomyopathy (7.6 vs. 6.4%, *P* = 0.024), COPD (9.5 vs. 4.5%, *P* < 0.001), renal insufficiency (10.5 vs. 6.9%, *P* < 0.001), mean age (61.58 vs. 58.17 years, *P* < 0.001), SBP (141.85 vs. 132.98 mmHg, *P* < 0.001), DBP (84.16 vs. 80.54 mmHg, *P* < 0.001), and HR (85.91 vs. 85.21 bpm, *P* = 0.008). There were no significant differences in mean body mass index (BMI) and proportion of drinkers between the two groups. Table 1 shows the characteristics of patients before and after PSM.

**TABLE 1:** Characteristics of the patients.

	Before PSM (*n* = 11,519)			After PSM (*n* = 6,466)		
	AF	Without AF	*P*	AF	Without AF	*P*
Number	3212	8307	−	2462	4004	−
Female (n, %)	1534 (47.8)	3773 (45.4)	0.025	1179 (47.9)	1854 (46.3)	0.225
Age (years)	61.58 ± 13.99	58.17 ± 14.21	< 0.001	59.63 ± 13.39	58.98 ± 13.41	0.059
BMI (kg/m^2)	23.62 ± 3.67	23.77 ± 3.79	0.055	23.66 ± 3.67	23.69 ± 3.75	0.751
SBP (mmHg)	141.85 ± 22.40	132.98 ± 19.77	< 0.001	141.27 ± 23.14	132.96 ± 19.83	< 0.001
DBP (mmHg)	84.16 ± 13.09	80.54 ± 11.78	< 0.001	84.44 ± 13.52	80.40 ± 11.84	< 0.001
Heart rate (bpm)	85.91 ± 13.00	85.21 ± 12.43	0.008	85.83 ± 12.42	85.27 ± 12.16	0.073
Hypertension (n, %)	2434 (75.8)	5104 (61.4)	< 0.001	1742 (70.8)	2356 (58.8)	< 0.001
Smoke (n, %)	895 (27.9)	2105 (25.3)	0.006	608 (24.7)	999 (25.0)	0.841
Alcohol (n, %)	336 (10.5)	844 (10.2)	0.658	248 (10.1)	400 (10.0)	0.948
Diabetes (n, %)	675 (21.0)	1464 (17.6)	< 0.001	445 (18.1)	737 (18.4)	0.763
Stroke (n, %)	524 (16.3)	916 (11.0)	< 0.001	268 (10.9)	454 (11.3)	0.602
Coronary artery disease (n, %)	947 (29.5)	2031 (24.4)	< 0.001	606 (24.6)	952 (23.8)	0.462
Heart failure (n, %)	893 (27.8)	1767 (21.3)	< 0.001	502 (20.4)	807 (20.2)	0.844
Valvular heart disease (n, %)	321 (10.0)	556 (6.7)	< 0.001	143 (5.8)	240 (6.0)	0.800
Cardiomyopathy (n, %)	245 (7.6)	534 (6.4)	0.024	148 (6.0)	250 (6.2)	0.746
COPD (n, %)	305 (9.5)	377 (4.5)	< 0.001	93 (3.8)	144 (3.6)	0.758
Renal insufficiency (n, %)	336 (10.5)	573 (6.9)	< 0.001	181 (7.4)	265 (6.6)	0.280

PSM, propensity score matching; AF, atrial fibrillation; BMI, body mass index; SBP, systolic blood pressure; DBP, diastolic blood pressure; COPD, chronic obstructive pulmonary disease.

Multivariate analysis showed that the following factors had a significant positive correlation with AF: female (OR = 1.18, 95% CI: 1.08–1.29), age (OR = 1.01, 95% CI: 1.01–1.02), HR (OR = 1.01, 95% CI: 1.00–1.01), HBP (OR = 1.88, 95% CI: 1.71–2.08), stroke (OR = 1.31, 95% CI: 1.15–1.48), valvular heart disease (OR = 1.53, 95% CI: 1.32–1.78), COPD (OR = 1.93, 95% CI: 1.63–2.29), and renal insufficiency (OR = 1.40, 95% CI: 1.21–1.63). Smoking (OR = 1.04, 95% CI: 0.94–1.16), diabetes (OR = 1.07, 95% CI: 0.96–1.19), and heart failure (OR = 1.10, 95% CI: 0.97–1.25) were positively correlated with AF, but not statistically significant. BMI, drinking, coronary artery disease and cardiomyopathy were not correlated with AF (Supplementary Figure 1).

### Sex differences in the relationship between hypertension and atrial fibrillation

**FIGURE 2:**
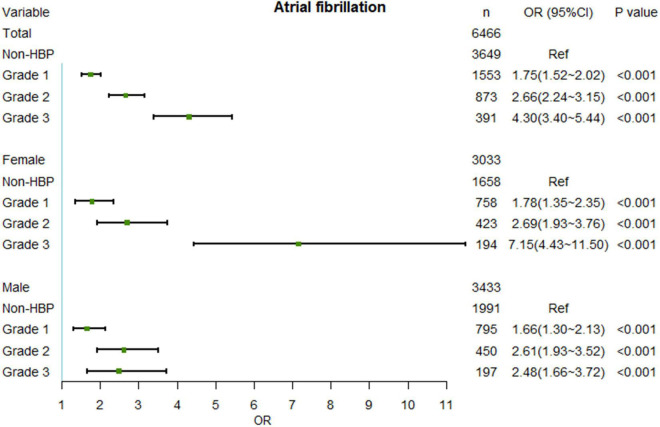
Relationship between AF and different HBP level after PSM. No interaction effects existed between sex and HBP levels. AF, atrial fibrillation; OR, odd ratio; HBP, hypertension; PSM, propensity score matching.

**FIGURE 3:**
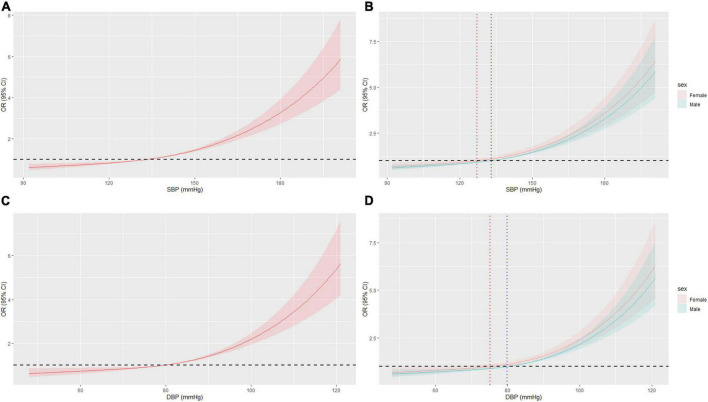
Restricted cubic spline (RCS) of BP and AF in all patients after PSM. **(A)** RCS of SBP and AF. **(B)** RCS of SBP and AF in different sex, OR = 1 when SBP = 127.1 mmHg or 133.1 mmHg in female or male, respectively. **(C)** RCS of DBP and AF. **(D)** RCS of DBP and AF in different sex, OR = 1 when DBP = 75.1 mmHg or 79.9 mmHg in female or male, respectively. BP, blood pressure; AF, atrial fibrillation; OR, odd ratio; PSM, propensity score matching.

Elevated BP was significantly highly correlated with AF risk. Compared with patients with normal BP, patients with grade 1 HBP had a 1.75 (95% CI: 1.52–2.02)-fold increase in the exposure ratio of AF, and in patients with grade 2 and 3 HBP, the ratio was further increased to 2.66 (95% CI: 2.24–3.15) and 4.30 (95% CI 3.40–5.44), respectively (Figure 2). Especially with the elevation of SBP, the OR of AF increased significantly (Figures 3A,C).

In the sex subgroup, the results maintained the same trend, but the relationship appeared to be stronger in female patients. While the risk of AF in females with grade 1 and 2 HBP was slightly higher than that in males, the OR of AF was significantly higher in females with grade 3 HBP than in males (OR = 7.15, 95% CI: 4.43–11.50 vs. OR = 2.48, 95% CI: 1.66–3.72) (Figure 2). Also, the BP cut-off for increased OR of AF was lower in females than in males (127.1/75.1 mmHg vs. 133.1/79.9 mmHg) (Figures 3B,D). The results before PSM are showed in Supplementary Figures 2, 3.

### Sex differences in the relationship between blood pressure controlled and atrial fibrillation

**FIGURE 4:**
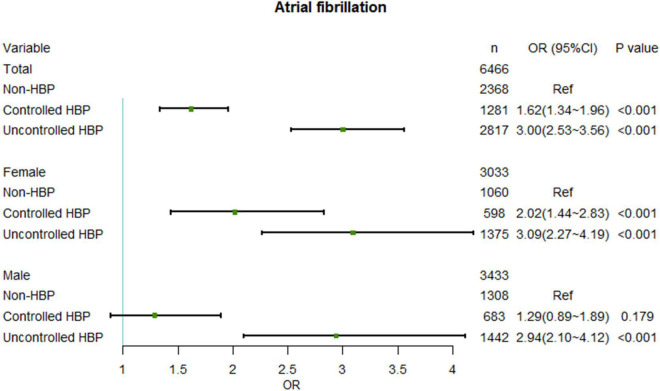
Relationship between AF and different BP controlled group after PSM. No interaction effects existed between sex and BP controlled groups. AF, atrial fibrillation; OR, odd ratio; BP, blood pressure; PSM, propensity score matching.

**FIGURE 5:**
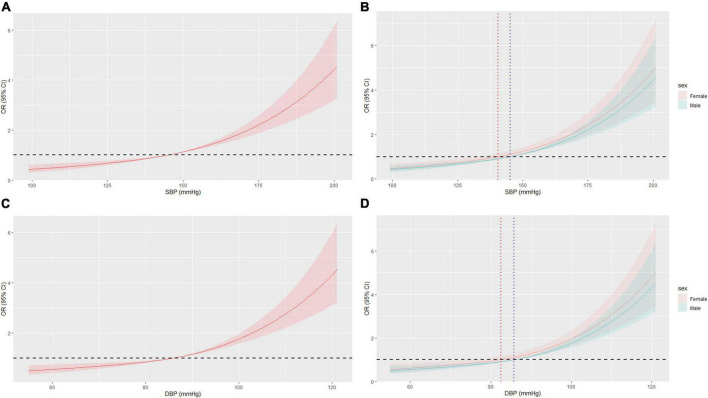
Restricted cubic spline (RCS) of BP and AF in HBP patients after PSM. **(A)** RCS of SBP and AF. **(B)** RCS of SBP and AF in different sex, OR = 1 when SBP = 140.5 mmHg or 145.1 mmHg in female or male, respectively. **(C)** RCS of DBP and AF. **(D)** RCS of DBP and AF in different sex, OR = 1 when DBP = 82.5 mmHg or 85.8 mmHg in female or male, respectively. AF, atrial fibrillation; BP, blood pressure; HBP, hypertension; OR, odd ratio; PSM, propensity score matching.

In HBP patients, BP uncontrolled was highly correlated with AF (OR = 3.00, 95% CI: 2.53–3.56), which appears to be more obvious in females (OR = 3.09, 95% CI: 2.27–4.19). However, with good BP control, the OR of AF was reversed in both sex subgroups. Compared with HBP uncontrolled patients, controlling HBP reduced the OR of AF in HBP controlled patients to 1.62 (95% CI: 1.34–1.96), in particular, controlling HBP reduced the OR of AF in females and males to 2.02 (95% CI: 1.44–2.83) and 1.29 (95% CI: 0.89–1.98), respectively (Figure 4). Furthermore, in HBP patients, lowering BP was obviously associated with reduced prevalence of AF (Figures 5A,C), with BP cut-offs of 140.5/82.5 mmHg and 145.1/85.8 mmHg for females and males, respectively (Figures 5B,D). The results before PSM are showed in Supplementary Figures 4, 5.

### Atrial fibrillation occurs earlier in females

**FIGURE 6:**
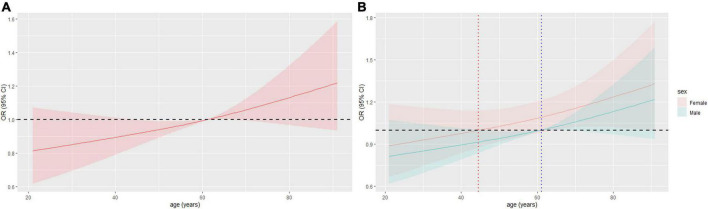
Restricted cubic spline (RCS) of age and AF in all patients after PSM. **(A)** RCS of age and AF. **(B)** RCS of age and AF in different sex, OR = 1 when age = 44.6 years or 61.1 years in female or male, respectively. AF, atrial fibrillation; OR, odd ratio; PSM, propensity score matching.

The prevalence of AF increased with age (Figure 6A). Females was more likely to have AF than males at a younger age, with a cut-off age of 44.6 years for females and 61.1 years for males (Figure 6B). Before PSM, the cut-off ages for females and males were 48.6 and 61.1 years, respectively (Supplementary Figure 6).

## Discussion

There is a strong relationship between HBP and AF, and the two share many common risk factors. HBP is also a key risk factor for AF. However, neither previous studies nor current guidelines clarify what high levels of BP should be controlled to reduce the occurrence of AF (13–16). In this study of a large case-control population, we provided a BP cut-off that was significantly associated with increased prevalence of AF and a cut-off that was associated with reduced prevalence of AF in hypertensive individuals. We demonstrated lower BP levels associated with an increased prevalence of AF in women than in men, indicating that women with HBP may need to control BP to a stringent target to reduce the prevalence of AF.

Studies have shown that multiple risk factors are closely related to the occurrence of AF, including uncontrollable factors such as age, sex, family history, race, height, and genetics (19, 22), controllable factors such as HBP, diabetes, myocardial infarction, valvular heart disease, COPD, chronic kidney disease, obesity, endurance exercise, sleep apnea, thyroid dysfunction, smoking, and intemperance (19, 22, 23), as well as some laboratory parameters such as left ventricular hypertrophy, left atrial enlargement, reduced left ventricular short axis shortening rate, C-reactive protein, and plasma brain natriuretic peptide (19). These findings are largely consistent with our results. After adjusting by multivariate regression, we observed that age, HBP, valvular heart disease, COPD, and renal insufficiency were risk factors for AF (Supplementary Figure 1). However, BMI, smoking, alcohol consumption, diabetes, and coronary artery disease were not significantly associated with AF risk (Supplementary Figure 1). This observation may be due to the nature of the patients we included. As all participants were from our cardiology unit, the risk factors we identified were highly related to most cardiovascular diseases. These risk factors may not differ between groups with and without AF in patients admitted for other cardiovascular diseases. Specifically for coronary disease, we excluded patients with acute myocardial infarction who are more likely to develop AF than those with common coronary heart disease (24, 25). It is worth mentioning that female gender appears to be a risk factor for AF in this study, unlike what we know (19). The reason may be that our study subjects were all patients, unlike a comprehensive community survey, which recruits healthy persons.

In the general population, the occurrence and recurrence of AF were associated with BP (26, 27). Even high normal BP is an important predictor of new-onset AF (6, 8). Our results confirm this. Compared with the non-HBP patients, the AF ORs of patients with grades 1, 2, and 3 HBP increased by 1.75, 2.66, and 4.30 times, respectively. The BP cut-off for significantly increasing the OR of AF was approximately 130/75 mmHg. Not only that, but the influence of BP on the risk of AF in females was more pronounced, especially when BP was significantly elevated. We demonstrated that the OR for AF in females with grade 3 HBP was 7.15 times higher than in non-hypertensive females. Meanwhile, the BP cut-off for females (127.1/75.1 mmHg) to develop AF was lower than that for males (133.1/79.9 mmHg), suggesting that more attention should be paid to women, even in the early stages of high normal BP.

In hypertensive patients, BP control can significantly reduce the incidence of AF. Compared with the non-hypertensive patients, the OR of AF in patients with uncontrolled BP was 3.00 times, which decreased to 1.62 times after BP control, fully demonstrating the importance of BP control. In hypertensive patients, lowing BP to 145.1/85.8 mmHg had a significant effect on the OR of AF, whereas in female patients, it should be lowered to 140.5/82.5 mmHg.

After BP control, the risk of AF decreases. On the one hand, the remodeling of the atrial myocardium by HBP is fundamentally released after BP reduction, (28). On the other hand, antihypertensive drugs such as angiotensin-converting enzyme inhibitors, angiotonin receptor blockers, and angiotensin receptor neprilysin inhibitor can significantly improve cardiac remodeling and reverse the structural basis of AF (29), which also help relieve AF.

Left atrial remodeling is the main cause of AF in hypertensive patients. Verdecchia et al. conducted a 16-year follow-up of 2,482 patients with primary HBP and found that the risk of AF increases with patient age and left ventricular size, and that increased left atrial volume predisposes to chronic AF (30). This is because elevated BP leads to an increase in left ventricular end-diastolic pressure, resulting in left ventricular hypertrophy and diastolic dysfunction, which in turn increase left atrial pressure. Left atrial remodeling induced by long-standing elevated left atrial pressure is the mechanistic basis of AF (31, 32). In animal models, Kistler et al. performed electrophysiological and pathological examinations on chronic hypertensive sheep and found that elevated BP was significantly positively correlated with atrial electrical and anatomical remodeling (33). Lau et al. also used a sheep model of HBP to study the effect of HBP on atrial remodeling and demonstrated that early antihypertensive therapy was effective in delaying this process (34). In a follow-up of 112 patients with AF, Tanabe et al. found that the left atrium was reversed in hypertensive patients with controlled BP compared with those with uncontrolled BP (35), supporting that BP control in hypertensive patients is of great significance.

Sex differences in the relationship of HBP and AF is the highlight of this study. On the one hand, women tend to have lower BP for reasons such as estrogen (36), weight loss, dieting, and bland diets. Many women have low BP but no clinic signs of shock. This means that normal or high normal BP may be a hypertensive state in women. Other possible reasons include perimenopause and post-menopause. Studies have found that perimenopausal and post-menopausal women are at increased risk of various cardiovascular diseases, especially early menopause, which is associated with estrogen (37–39). In this study, we added to analyze age as a sex-disaggregated risk factor for AF and found that the OR of AF increased significantly in women after 44.6 or 48.6 years (Figure 6; Supplementary Figure 6), which roughly coincides with the timing of menopause in women, especially those with earlier menopause. Relatively, the cut-off age was older for men. However, further studies are needed to consider information such as the timing of menopause.

In this study, to minimize the information loss caused by data grouping, in addition to logistic regression, we also conducted RCS to directly process the data. The RCS results showed that unexpectedly, the BP cut-off values for a significantly increased OR of AF due to elevated BP and a reduced OR of AF after BP control in hypertensive patients were not 140/90 mmHg and 130/80 mmHg, respectively (40), and there were significant differences in the cut-off value of males and females. Meanwhile, the results before and after PSM were basically the same, indicating the stability of the data.

This study has some limitations. First, the collected data came from a single-center retrospective study, and further verification is needed in prospective studies with larger samples and more centers. Second, this study did not collect data on echocardiogram, HBP medication, and menstruation and menopause status of female patients. The interpretation of relevant conclusions is limited to our current findings and previous literature reports, and further confirmation is needed in follow-up work. Overall, however, these do not affect the clinical significance of our findings, which convey to clinicians that BP targets for AF risk reduction should include the sex factor.

## Conclusion

Blood pressure (BP) over 133.1/79.9 mmHg in male patients or 127.1/75.1 mmHg in female patients was significantly associated with the prevalence of AF. In HBP patients, a BP lower than 145.1/85.8 mmHg in men or 140.5/82.5 mmHg in women was associated with reduced prevalence of AF. These data suggest that BP targets to reduce the risk of AF may consider sex differences, and that a lower and individualized BP target may be established to protect against AF in women.

## Data availability statement

The datasets used and analyzed during the current study are available from the corresponding author on reasonable request. Requests to access these datasets should be directed to XT, tangxh007007@163.com.

## Ethics statement

The studies involving human participants were reviewed and approved by IRB of The Third Xiangya Hospital of Central South University. Written informed consent for participation was not required for this study in accordance with the national legislation and the institutional requirements.

## Author contributions

XZ and QF established the idea and helped in wrote main ideas for this research, main results, and discussion of the findings. AW was a major contributor in wrote the manuscript. XZ and WJ interpreted statistical analysis to prove the main findings of this project. QF, XW, and JW collected data. XT contributed on editing this manuscript and giving advice for the main authors to organized the manuscript and ideas of the project. All authors contributed to the article and approved the submitted version.
