# Supplementary material for: Sex differences in the association between blood pressure and atrial fibrillation: A case-control study
Source: Front Cardiovasc Med. 2022 Dec 8;9:1061240. doi: 10.3389/fcvm.2022.1061240 (PMC9772049; doi:10.3389/fcvm.2022.1061240)
Supplement: Supplementary file 1 [file Data_Sheet_1.DOCX]

Supplementary Material

**Contents**

Supplementary Figure 1. Risk factors of atrial fibrillation.

Supplementary Figure 2. Relationship between AF and different HBP level before PSM.

Supplementary Figure 3. RCS of BP and Af in all patients before PSM.

Supplementary Figure 4. Relationship between AF and different BP controlled group before PSM.

Supplementary Figure 5. Restricted cubic spline (RCS) of BP and AF in HBP patients before PSM.

Supplementary Figure 6. Restricted cubic spline (RCS) of age and AF in all patients before PSM.


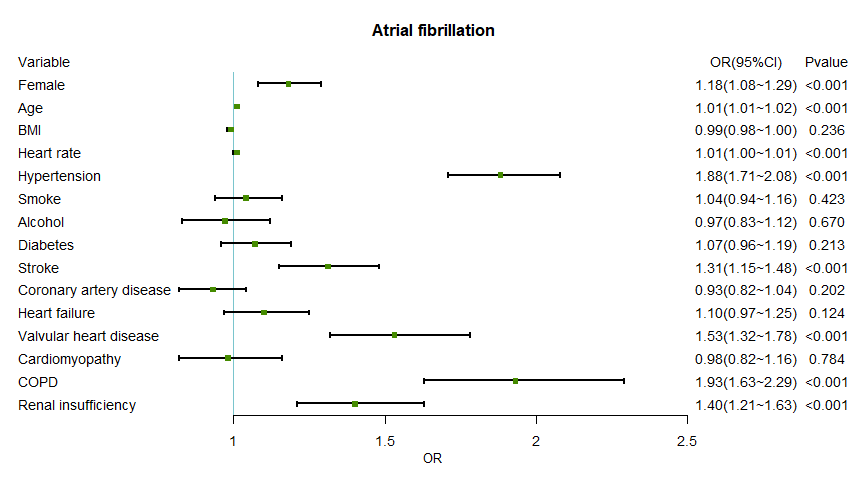


Supplementary Figure 1. Risk factors of atrial fibrillation before propensity score matching.


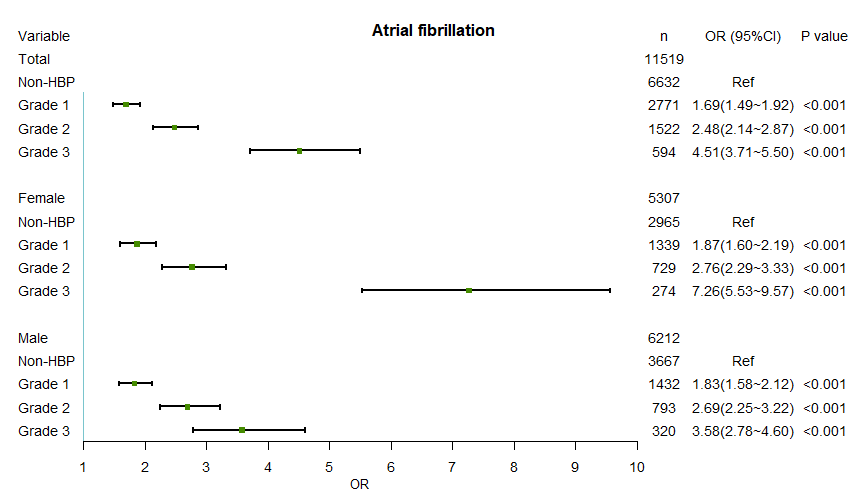


Supplementary Figure 2. Relationship between AF and different HBP level before PSM. Logistic regression model was adjusted by sex, age, BMI, HR, smoke, alcohol, diabetes, stroke, coronary artery disease, heart failure, valvular heart disease, cardiomyopathy, COPD and renal insufficiency. No interaction effects existed between sex and HBP levels. AF, atrial fibrillation; OR, odd ratio; HBP, hypertension; PSM, propensity score matching.

| a | b |
| --- | --- |
| 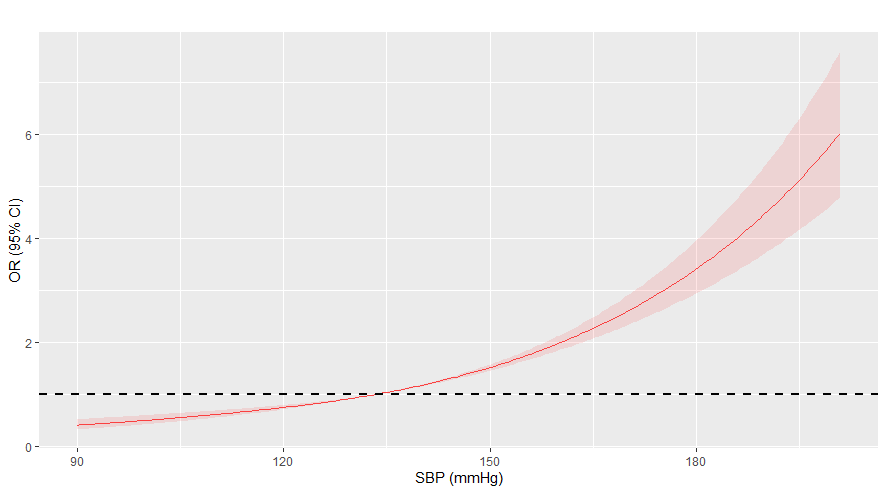 | 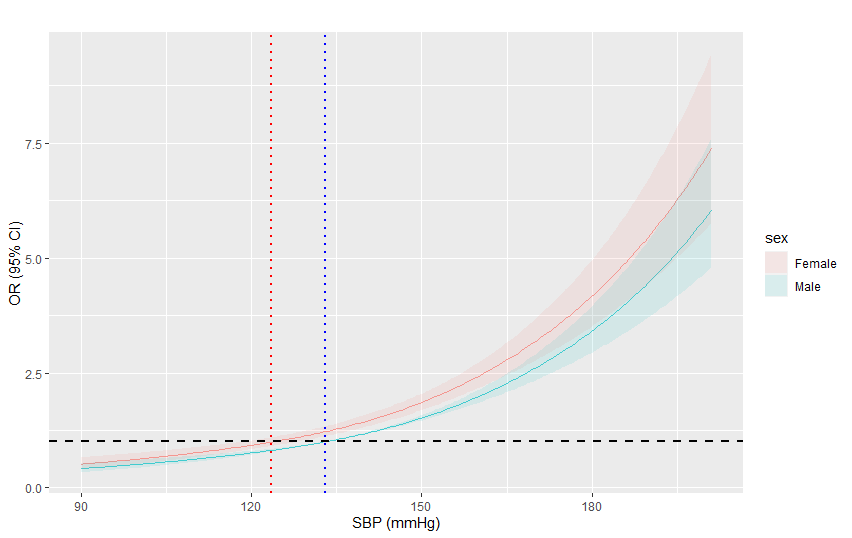 |
| c | d |
| 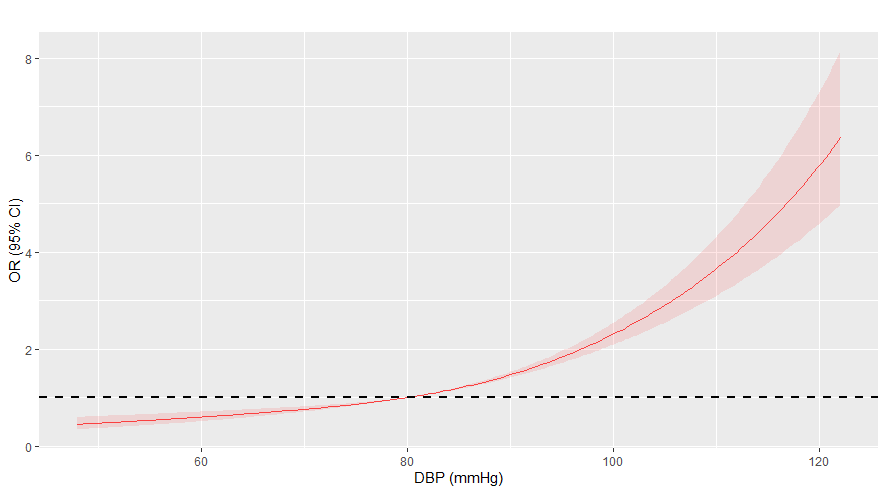 | 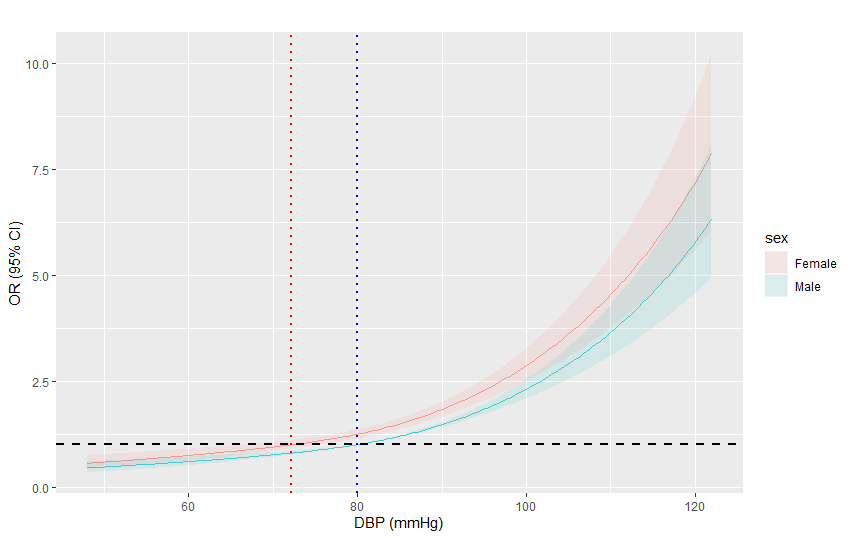 |

Supplementary Figure 3. RCS of BP and AF in all patients before PSM. Models were adjusted by sex, age, BMI, HR, smoke, alcohol, diabetes, stroke, coronary artery disease, heart failure, valvular heart disease, cardiomyopathy, COPD and renal insufficiency. a) RCS of SBP and AF; b) RCS of SBP and AF in different sex, OR=1 when SBP=123.5mmHg or 133.0mmHg in female or male, respectively; c) RCS of DBP and AF; d) RCS of DBP and AF in different sex, OR=1 when DBP=72.2mmHg or 80.0mmHg in female or male, respectively. RCS, restricted cubic spline; PSM, propensity score matching.


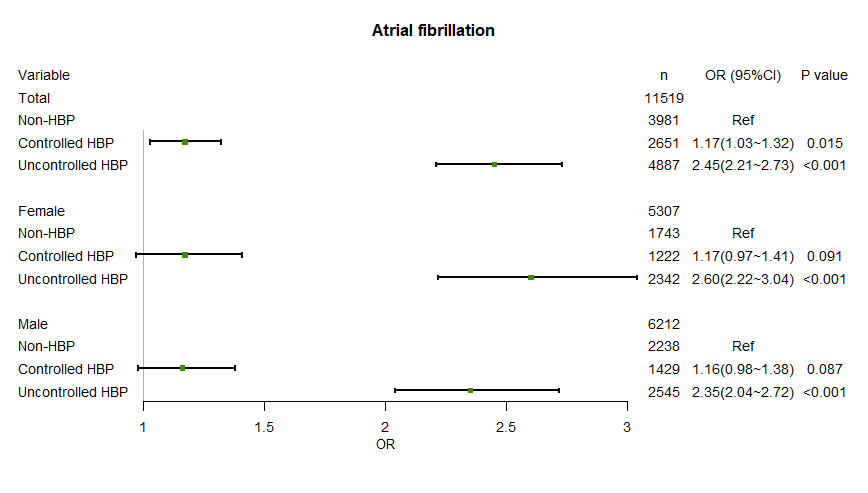


Supplementary Figure 4. Relationship between AF and different BP controlled group before PSM. Logistic regression model was adjusted by sex, age, BMI, HR, smoke, alcohol, diabetes, stroke, coronary artery disease, heart failure, valvular heart disease, cardiomyopathy, COPD and renal insufficiency. No interaction effects existed between sex and BP controlled groups. AF, atrial fibrillation; OR, odd ratio; BP, blood pressure; PSM, propensity score matching.

| a | b |
| --- | --- |
| 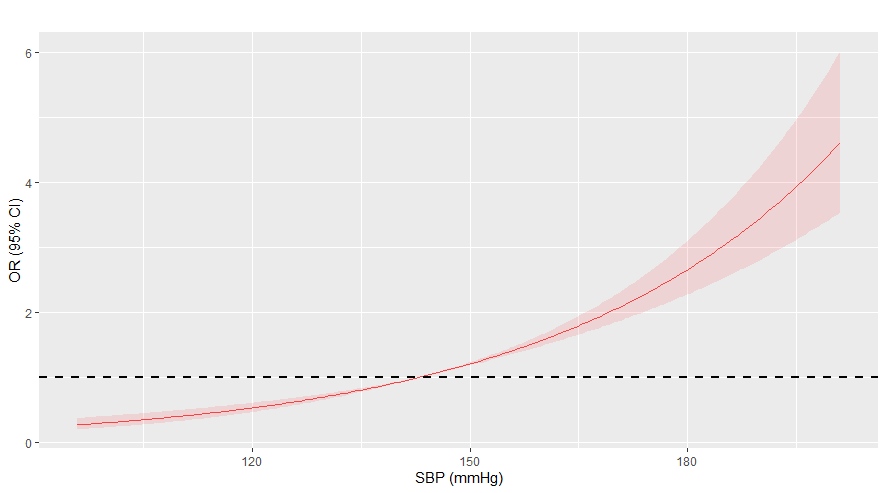 | 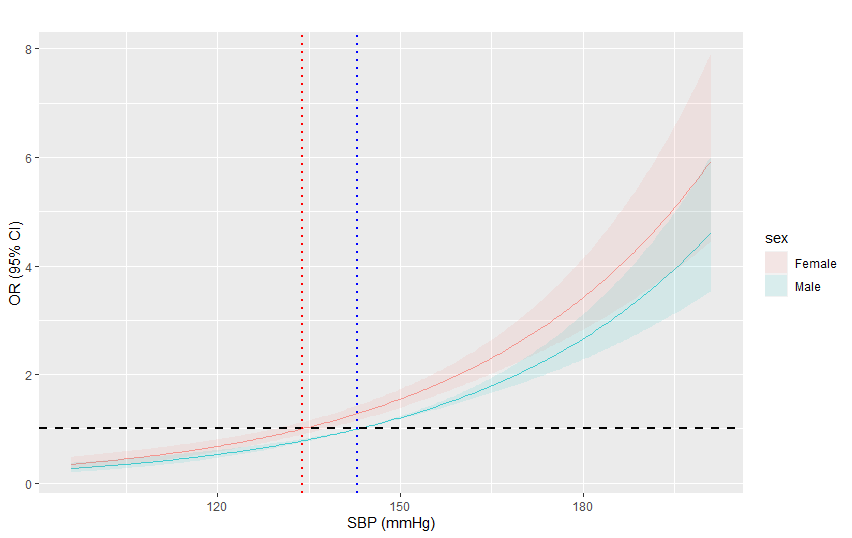 |
| c | d |
| 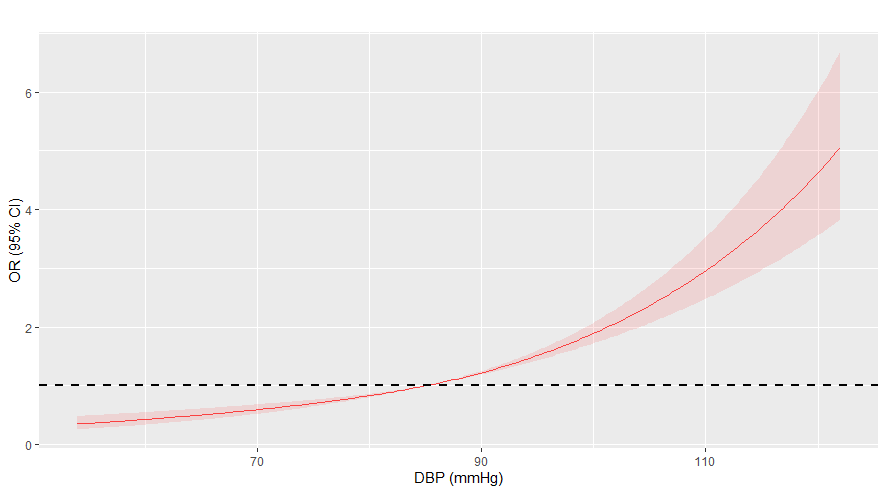 | 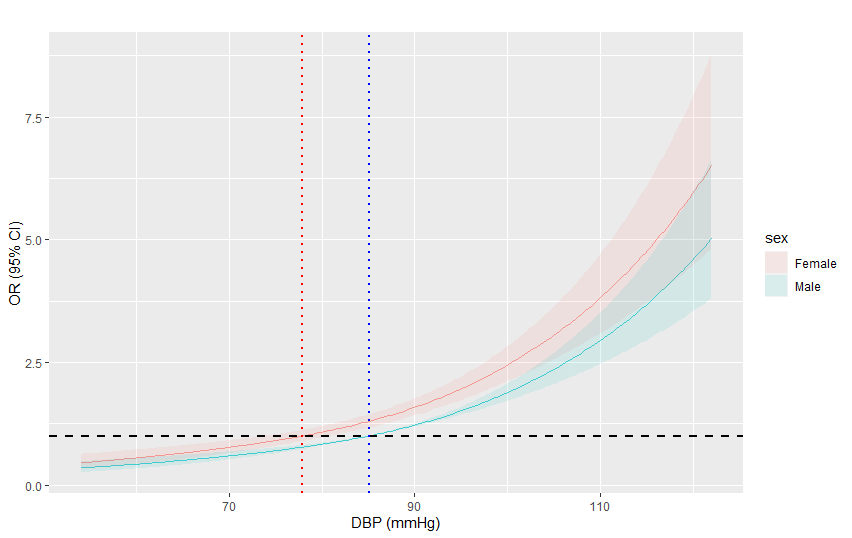 |

Supplementary Figure 5. Restricted cubic spline (RCS) of BP and AF in HBP patients before PSM. Models were adjusted by sex, age, BMI, HR, smoke, alcohol, diabetes, stroke, coronary artery disease, heart failure, valvular heart disease, cardiomyopathy, COPD and renal insufficiency. a) RCS of SBP and AF; b) RCS of SBP and AF in different sex, OR=1 when SBP=134.0mmHg or 143.0mmHg in female or male, respectively; c) RCS of DBP and AF; d) RCS of DBP and AF in different sex, OR=1 when DBP=77.9mmHg or 85.1mmHg in female or male, respectively. AF, atrial fibrillation; BP, blood pressure; HBP, hypertension; OR, odd ratio; PSM, propensity score matching.

| a | b |
| --- | --- |
| 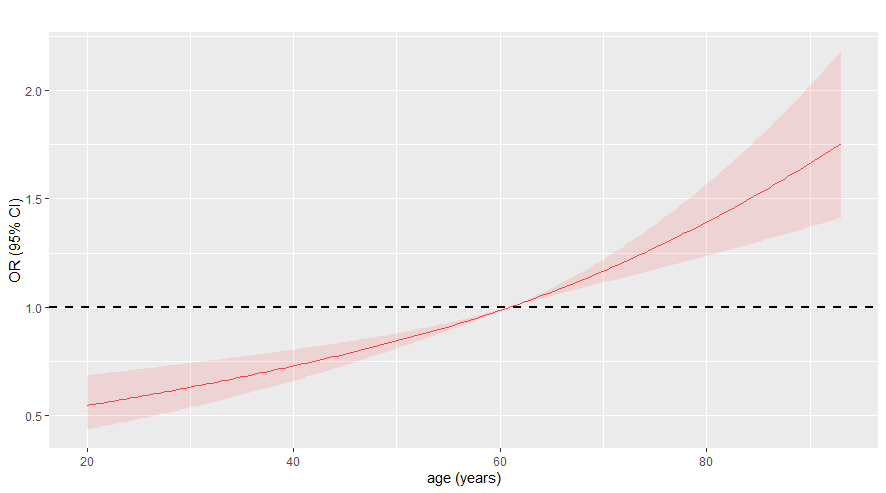 | 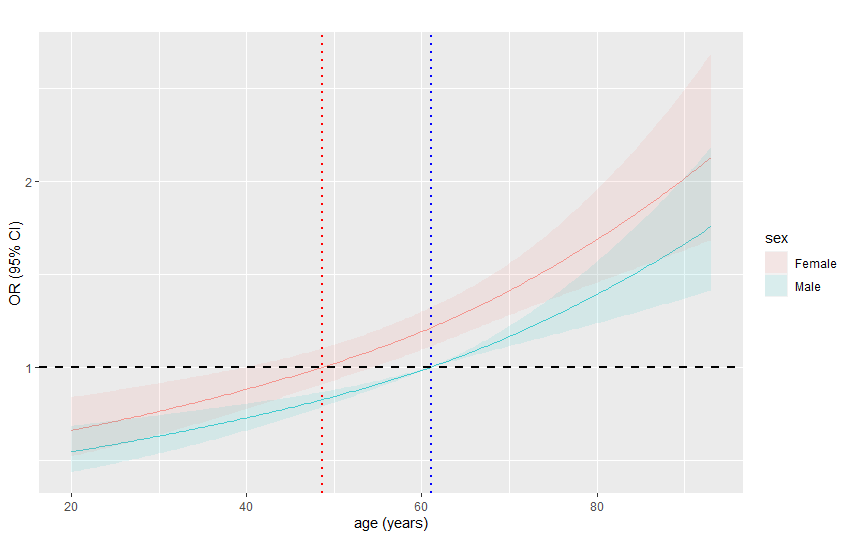 |

Supplementary Figure 6. Restricted cubic spline (RCS) of age and AF in all patients before PSM. Models were adjusted by sex, BMI, HR, smoke, alcohol, diabetes, stroke, coronary artery disease, heart failure, valvular heart disease, cardiomyopathy, COPD and renal insufficiency. a) RCS of age and AF; b) RCS of age and AF in different sex, OR=1 when age=48.6 years or 61.1 years in female or male, respectively. AF, atrial fibrillation; OR, odd ratio; PSM, propensity score matching.
